# PVR Mediates Resistance to IL21.CD276.CAR-T Therapy in Esophageal Squamous Cell Carcinoma

**DOI:** 10.3390/ijms27156725

**Published:** 2026-07-28

**Authors:** Lihong Wang, Qijing Guo, Wenkai Han, Xiaoxuan Tao, Tong Ye, Li Sun, Yiming Gao, Anna Niu, Hui Zhao, Xiaoyan Liu, Yu Wang

**Affiliations:** 1 Department of Oncology, Air Force Medical Center, People’s Liberation Army, Beijing 100142, China; lhwang2017@163.com (L.W.);; 2 School of Medicine, Northwest University, Xi’an 710069, China; 3 Department of Pharmacy, Air Force Medical Center, People’s Liberation Army, Beijing 100036, China; 4 Department of Biomedical Engineering, City University of Hong Kong, Kowloon, Hong Kong SAR 999077, China

**Keywords:** esophageal squamous cell carcinoma, CD276, CAR-T cells, IL-21, poliovirus receptor, drug resistance

## Abstract

Chimeric antigen receptor (CAR)-T therapy has achieved partial therapeutic efficacy in solid tumors, but its overall effectiveness remains limited. IL-21-armored CD276.CAR-T (IL21.CD276.CAR-T) represents a promising strategy to enhance anti-tumor activity against esophageal squamous cell carcinoma (ESCC). However, resistance mechanisms remain unclear. We generated IL21.CD276.CAR-T cells and evaluated their cytotoxicity *in vitro* and in B-NDG xenograft models. Poliovirus receptor (PVR) expression on ESCC cells was analyzed, and shRNA-mediated *PVR* knockdown was performed to validate its role in resistance. IL-21R expression on ESCC cells was examined to exclude direct IL-21 signaling. IL-21 enhances the cytotoxicity of CD276.CAR-T cells against ESCC. Although IL21.CD276.CAR-T exhibited potent cytotoxicity *in vitro*, it failed to achieve complete tumor regression, and resistant cells still emerged. Mechanistically, resistance was not caused by CD276 antigen loss or IL-21R expression, but by upregulation of PVR on cancer cell membrane after IL21.CAR-T exposure. *PVR* knockdown restored CAR-T sensitivity *in vitro*, enhanced the anti-tumor capacity of IL21.CD276.CAR-T cells, and partially improved anti-tumor efficacy *in vivo* without systemic toxicity. PVR may act as a mediator of resistance to IL21.CD276.CAR-T in ESCC. Targeting PVR represents a novel strategy to overcome IL21.CAR-T resistance and improve therapeutic outcomes in ESCC.

## 1. Introduction

Esophageal squamous cell carcinoma (ESCC), which accounts for more than 90% of all esophageal malignancies, represents a highly prevalent malignant tumor worldwide, characterized by aggressive invasiveness and extremely dismal clinical outcomes [1]. Despite the availability of chemotherapy, radiotherapy, targeted therapy and immunotherapy, the 5-year survival rate for patients with advanced ESCC remains low, highlighting the urgent need for more effective treatment strategies [2]. Due to the frequent deletion, downregulation and single nucleotide polymorphisms of major histocompatibility complex (MHC) class I molecules in Chinese ESCC patients, CAR-T therapy is particularly suitable for this disease, as its tumor antigen recognition is independent of MHC restriction [3]. CD276 (B7-H3) is a tumor-associated immune checkpoint molecule highly expressed in ESCC, prostate cancer, hepatocellular cancer, colon adenocarcinoma and other multiple solid tumors, while showing low or absent expression in most normal tissues [4,5,6,7]. Beyond immune suppression, CD276 promotes tumor proliferation, metastasis, metabolic reprogramming, and therapeutic resistance, making it a functionally critical driver of cancer progression [8]. Its tumor-predominant expression pattern and key roles in malignant progression render CD276 an ideal and safe target for CAR-T therapy, with high tumor specificity and minimal on-target off-tumor toxicity [9].

Currently, several early-phase clinical trials have been conducted using CD276-targeted CAR-T cells in patients with glioblastoma, pediatric solid tumors, and the results have demonstrated manageable safety profiles and preliminary clinical responses including partial remissions, yet durable efficacy remains limited [10,11]. The therapeutic efficacy of CAR-T cells in solid tumors is severely limited by multiple critical obstacles, including the dense physical barrier of the tumor microenvironment, insufficient T cell infiltration, severe T cell exhaustion, the immunosuppressive microenvironment, and the rapid emergence of acquired resistance, all of which ultimately lead to treatment failure [12].

To overcome these limitations and enhance the anti-tumor function and persistence of CAR-T cells, armoring with interleukin-21 (IL-21) has emerged as a promising strategy. IL-21 is one of the common γ-chain cytokines. It is mainly secreted by CD4^+^ T cells, especially follicular helper T (Tfh)-like cells and can regulate a variety of immune cell subsets, including T cells, B cells, natural killer cells, macrophages, dendritic cells, and others [13,14,15,16]. Some researchers have found that IL-21 alone or in combination with IL-7, IL-15, C-X-C motif chemokine ligand 9 can enhance CAR-T anti-tumor function targeting epidermal growth factor receptor (EGFR), natural killer group 2D (NKG2D), glypican-3 (GPC3) and other molecules in preclinical trials of esophageal cancer, liver cancer and other tumors [17,18,19]. However, both these studies and our preliminary research have revealed that drug-resistant cells still emerge after IL21.CAR-T-mediated tumor killing, and the specific underlying mechanism remains to be investigated.

After murine T cells were co-incubated with IL-21 for 48 h, RNA sequencing showed elevated expression levels of *TIGIT* (T cell immunoglobulin and ITIM domain), *Lag3*, *Pdcd1*, *Havcr2*, and *Cd244* [20]. TIGIT is a co-inhibitory receptor discovered in 2009 that is expressed on the surface of activated T and NK cells. It contains an extracellular IgV domain and a short intracellular region harboring an immunoreceptor tyrosine-based inhibitory motif (ITIM). TIGIT has two ligands, PVR (CD155) and PVRL2 (CD112), with PVR being the primary one, which mediates negative immune regulatory functions [21,22]. Upon PVR binding, the phosphorylated ITIM motif of TIGIT recruits SHP2 phosphatase (pSHP2) to transmit intracellular inhibitory signals and abrogate TCR/CAR proximal activation signaling such as ZAP70 phosphorylation (pZAP70). Recent evidence has revealed that the TIGIT/PVR inhibitory axis is closely associated with T cell exhaustion and apoptosis [23]. Based on the above findings, we hypothesized that the upregulation of the TIGIT/PVR pathway may participate in the acquisition of resistance to IL-21-armed CAR-T cells.

Here, we constructed CD276.CAR-T and IL-21-armed CD276.CAR-T cells for targeting ESCC, and found that their cytotoxicity both *in vitro* and *in vivo* was superior to that of conventional CD276.CAR-T cells, yet resistant cells still emerged. To elucidate the mechanism underlying resistance in IL21.CD276.CAR-T cells, we intend to simulate a resistant tumor microenvironment and examine changes in PVR expression on the membrane of ESCC cells. Furthermore, by regulating PVR expression, we will verify *in vitro* and *in vivo* whether PVR is involved in the resistance of IL21.CD276.CAR-T cells, so as to provide a theoretical basis for promoting the early clinical application of CAR-T cells in the treatment of solid tumors.

## 2. Results

### 2.1. Construction and Function of CD276.CAR-T and IL21.CD276.CAR-T Cells

**Figure 1:**
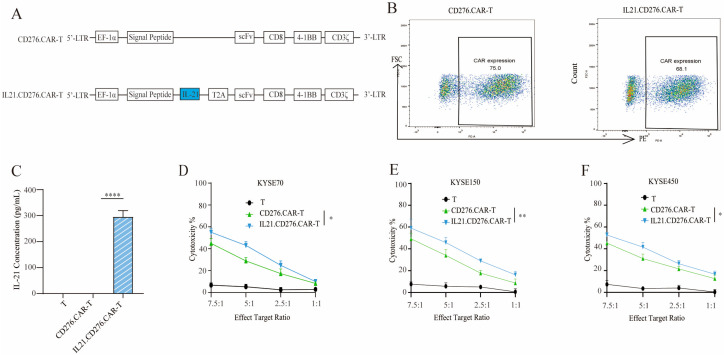
Generation and function of IL-21-armored CD276.CAR-T cells. (**A**) Schematic diagram of the lentiviral vectors encoding CD276.CAR and IL21.CD276.CAR. (**B**) Flow cytometry (FCM) analysis of CAR expression on the membrane of CD276.CAR-T and IL21.CD276.CAR-T cells using Protein L. (**C**) ELISA of IL-21 secretion in the supernatants of T cells and CAR-T cells (one-way ANOVA). (**D**–**F**) Cytotoxicity of T cells and CAR-T cells against ESCC cell lines KYSE70 (**D**), KYSE150 (**E**), and KYSE450 (**F**) at different effector to target (E:T) ratios, assessed by LDH release assay (*n* = 3, One-way ANOVA with Tukey’s post-hoc test). * *p* < 0.05, ** *p* < 0.01, **** *p* < 0.0001.

In our previous study, we successfully generated CD276-targeted CAR-T cells and demonstrated their potent cytotoxicity against ESCC cells. To enhance the anti-tumor activity of CD276.CAR-T cells, we introduced the human IL-21 followed by *T2A* downstream of the signal peptide but upstream of the scFv domain (Figure 1A). Lentiviruses were produced and used to transduce human T cells at a multiplicity of infection (MOI) of 1.5–2. The expression of CAR on the membrane of CD276.CAR-T and IL21.CD276.CAR-T cells was detected using Protein L within 24 h prior to the experiment. As shown in Figure 1B, the CAR expression all exceeded 60%. Supernatants from T cells, CD276.CAR-T cells, and IL21.CD276.CAR-T cells were collected, and the concentration of IL-21 was measured by ELISA. T cells and CD276.CAR-T cells secreted negligible amounts of IL-21, whereas IL21.CD276.CAR-T cells efficiently secreted IL-21 into the supernatant at approximately (295 ± 24) pg/mL (Figure 1C).

We subsequently evaluated the cytotoxicity of T cells, CD276.CAR-T cells, and IL21.CD276.CAR-T cells against ESCC cell lines, KYSE70, KYSE150 and KYSE450 by LDH release assay. The results revealed that IL-21 significantly augmented the cytotoxic activity of CD276.CAR-T cells, and this enhancement was statistically significant (Figure 1D–F).

### 2.2. Resistance to IL21.CD276.CAR-T Cells Is Not Associated with CD276 Antigen Loss

**Figure 2:**
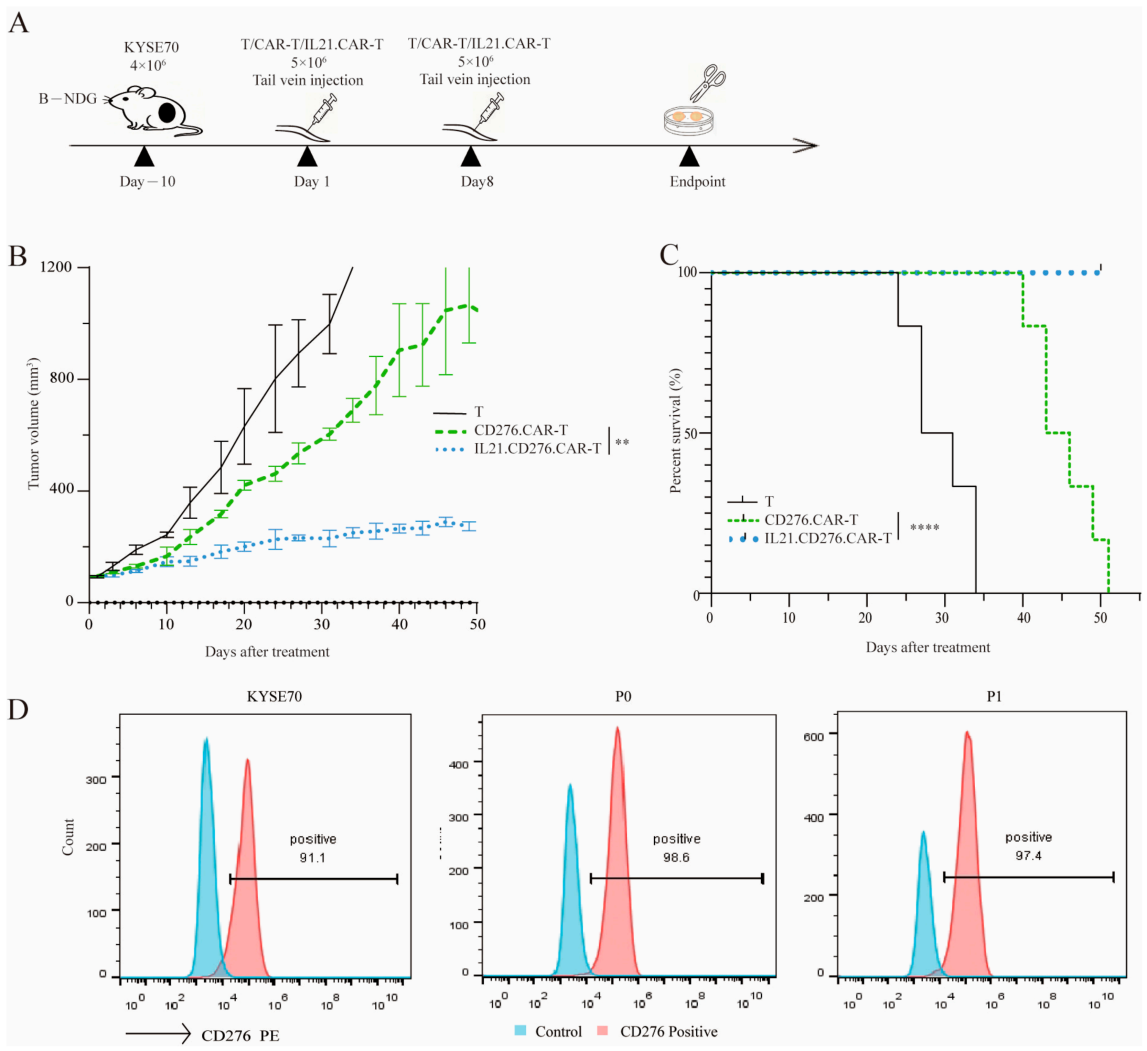
*In vivo* anti-tumor efficacy of IL21.CD276.CAR-T cells and validation of CD276 antigen expression in resistant tumor cells. (**A**) Schematic diagram of the B-NDG mouse xenograft model and treatment schedule. (**B**) Tumor growth curves of mice treated with T cells, CD276.CAR-T cells, or IL21.CD276.CAR-T cells (*n* = 6, one-way ANOVA). (**C**) Survival curves of mice in the three treatment groups (Log-rank test). (**D**) FCM analysis of CD276 antigen expression in parental KYSE70 cells and primary tumor cells isolated from residual tumors of the IL21.CD276.CAR-T group (P0), as well as after a second round of *in vitro* IL21.CD276.CAR-T killing (P1, *n* = 6). ** *p* < 0.01, **** *p* < 0.0001.

To evaluate the *in vivo* anti-tumor function of IL21.CD276.CAR-T cells, we established a KYSE70 xenograft model in B-NDG mice (Figure 2A). Tumor-bearing mice were treated with T cells, CD276.CAR-T cells, or IL21.CD276.CAR-T cells. While both CAR-T cell therapies significantly inhibited tumor growth compared to the T cells group, the tumors in both CD276.CAR-T and IL21.CD276.CAR-T groups continued to grow, and complete remission was not achieved (Figure 2B). Consistently, the survival curves showed that mice in the IL21.CD276.CAR-T group had significantly prolonged survival compared to the T and CD276.CAR-T groups (*p* < 0.0001) (Figure 2C).

To investigate whether CD276 antigen loss was responsible for resistance of IL21.CD276.CAR-T cells, we isolated primary tumor cells (P0) from residual tumors in the IL21.CD276.CAR-T group, and subjected them to a second round of IL21.CD276.CAR-T killing *in vitro* (P1). FCM analysis showed that CD276 expression remained high in both P0 (98.6%) and P1 (97.4%) cells’ membrane, similar to the parental KYSE70 cells (91.1%, Figure 2D). These results rule out CD276 antigen loss as the mechanism of resistance to IL21.CD276.CAR-T therapy. Further investigation into the mechanisms underlying resistance to IL21.CD276.CAR-T cells is therefore warranted.

### 2.3. PVR Is Involved in the Resistance of ESCC to IL21.CD276.CAR-T Cell-Mediated Cytotoxicity

**Figure 3:**
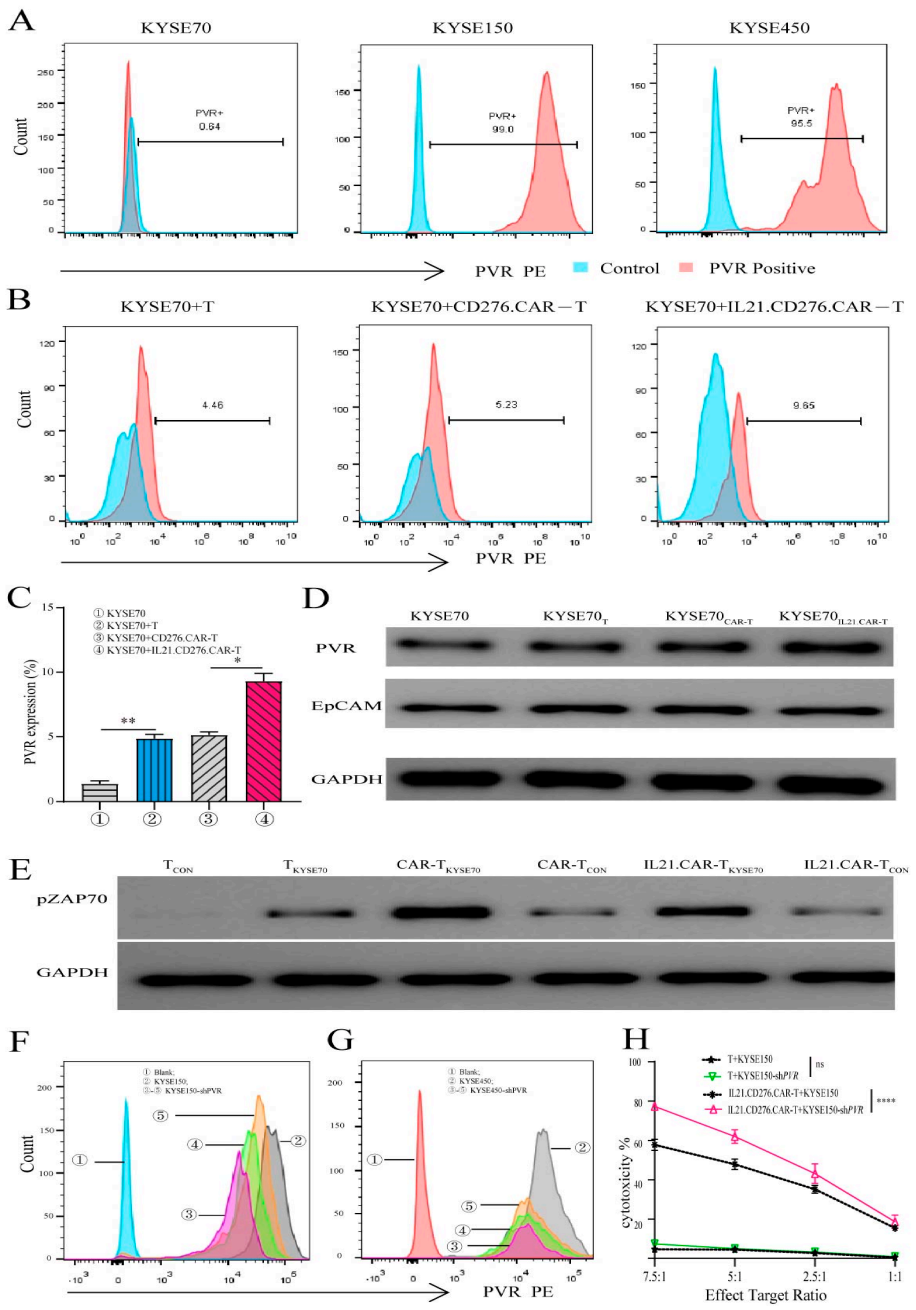
PVR mediates resistance to IL21.CD276.CAR-T cells. (**A**) FCM analysis of baseline PVR expression in KYSE70, KYSE150, and KYSE450. (**B**) PVR expression upregulated on KYSE70 cells after co-culture with T cells, CD276.CAR-T cells, or IL21.CD276.CAR-T cells (*n* = 3). (**C**) Quantification of PVR expression levels (One-way analysis of variance (one-way ANOVA) followed by Tukey’s multiple comparison test for pairwise comparisons between groups). (**D**) Western blot analysis of PVR and EpCAM expression in KYSE70 tumor cells after co-culture with T cells or CAR-T cells. GAPDH served as the internal loading control for protein normalization. (**E**) Western blot analysis of pZAP70 expression in T cells and CAR-T cells after co-culture with KYSE70 cells. GAPDH served as the internal loading control for protein normalization. (**F**,**G**) FCM validation of *PVR* knockdown efficiency in KYSE150 (**F**) and KYSE450 (**G**) cells. (**H**) LDH release assay showing the cytotoxic activity of T cells or IL21.CD276.CAR-T cells against parental KYSE150 cells or KYSE150-shPVR cells. Statistical comparisons between groups were performed using one-way ANOVA followed by Tukey’s multiple comparison test. * *p* < 0.05, ** *p* < 0.01, **** *p* < 0.0001, ns: not significant, CON: control.

To clarify whether PVR is involved in the resistance of ESCC to IL21.CD276.CAR-T cell therapy, we first examined the expression of PVR in these three ESCC cell lines, KYSE70, KYSE150 and KYSE450. We found that PVR expression was relatively low in KYSE70 cells, but high in both KYSE150 and KYSE450 cells (Figure 3A). Using the KYSE70 cell line (which exhibits low PVR expression), we established an IL21.CD276.CAR-T-resistant model by co-culturing cancer cells with CAR-T cells at an E:T of 1:1 for continuous 48h. After removal of suspended T or CAR-T cells, residual adherent KYSE70 cancer cells were harvested and subjected to FCM to detect membrane PVR expression. Subsequent FCM analysis revealed that, under the IL21.CD276.CAR-T-resistant condition, PVR expression on the membrane of ESCC cells increased from approximately 5% to (9.31 ± 0.60) % (Figure 3B,C, *p* < 0.05). We performed western blot (WB) to verify the alterations in PVR protein expression in KYSE70 cells before and after co-culture with T cells, CAR-T cells, and IL21.CAR-T cells (Figure 3D). GAPDH was used as a housekeeping internal reference protein to normalize the total protein loading amount of each sample lane and eliminate quantification bias caused by unequal protein loading. Compared with untreated KYSE70 cells, KYSE70 cells co-cultured with T or CAR-T cells exhibited elevated PVR levels, with the highest PVR expression observed in KYSE70 cells co-incubated with IL21.CAR-T cells. To rule out that the upregulation of PVR stemmed from experimental artifacts, we further examined the expression of epithelial cell adhesion molecule (EpCAM) in KYSE70 cells pre- and post-co-culture via WB, and no significant difference in EpCAM abundance was detected among all groups. To characterize the activation status of T, CAR-T, and IL21.CAR-T cells following co-culture with KYSE70 cells, cell lysates were harvested and subjected to WB analysis to quantify changes in pZAP70 levels (Figure 3E). We found that antigen stimulation increased intracellular pZAP70 in both T and CAR-T cells, with the most robust elevation detected in CAR-T cells. Three independent shRNA constructs were used to generate stable *PVR*-knockdown cell lines (Figure S1A), and single-cell clones were isolated. The knockdown efficiency was validated by FCM, showing varying degrees of *PVR* downregulation among different clones (Figure 3F,G). The KYSE150 clone with the most efficient *PVR* knockdown was selected for further functional assays. Consistent with our hypothesis, LDH release assays demonstrated that *PVR* knockdown significantly enhanced the cytotoxic activity of IL21.CD276.CAR-T cells against ESCC cells (Figure 3H, *p* < 0.0001). Furthermore, to exclude the possibility that IL-21 induces PVR upregulation by directly binding to IL-21R on tumor cells, we examined the surface expression of IL-21R in three ESCC cell lines (Figure S1B). The results showed that IL-21R was nearly undetectable in all three cell lines. These findings indicate that IL-21 does not regulate PVR expression through direct action on cancer cells.

### 2.4. The Prognostic Value, Diagnostic Value, and Immune Cell Infiltration of PVR in Esophageal Cancer

**Figure 4:**
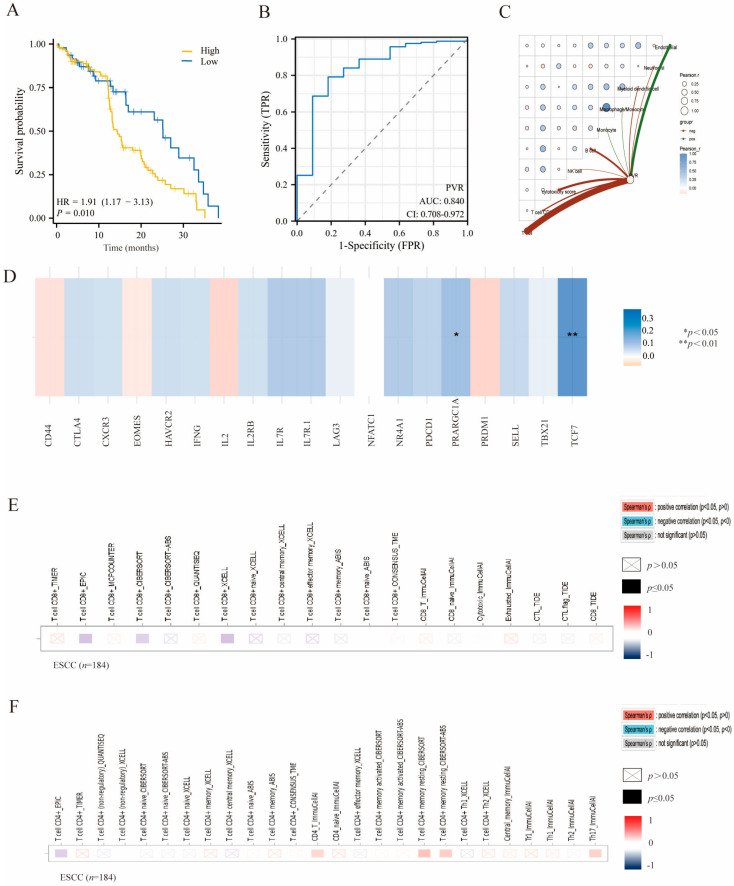
Clinical relevance of PVR expression and its correlation with the tumor immune microenvironment in ESCC. (**A**) Kaplan–Meier survival analysis of ESCC patients stratified by PVR expression (*n* = 184, Log–rank test). (**B**) Receiver operating characteristic (ROC) curve analysis evaluating the diagnostic performance of PVR in ESCC. (**C**) Correlation network analysis of immune cell infiltration in ESCC using six algorithms (Spearman’s rank correlation test). (**D**) Spearman’s correlation analysis between PVR expression and immune-related genes in ESCC. The color gradient represents the correlation coefficient, and asterisks indicate statistical significance. (**E**,**F**) Correlation heatmaps of PVR expression with immune cell signatures in ESCC, analyzed using the TIMER 3.0 database. Spearman’s rank correlation test was used to calculate correlation coefficients and corresponding *p* values. * *p* < 0.05, ** *p* < 0.01.

To explore the clinical significance of PVR in ESCC, we analyzed TCGA ESCC patient data. Kaplan–Meier survival analysis revealed that high PVR expression was significantly associated with poorer overall survival (HR = 1.91, 95% CI: 1.17–3.13, *p* = 0.010; Figure 4A). Receiver operating characteristic (ROC) curve analysis demonstrated that PVR expression had good diagnostic accuracy for ESCC (AUC = 0.840, 95% CI: 0.708–0.972; Figure 4B). As shown in the immune correlation network, PVR expression exhibited a significant negative correlation with T (r = 0.253, *p* < 0.01) cells and B cells, whereas it was positively correlated with immunosuppressive myeloid populations, including monocytes (r = 0.065, *p* < 0.05) and macrophages (r = 0.045, *p* < 0.05, Figure 4C). These correlative bioinformatic results indicate an association between high PVR levels and an immunosuppressive tumor microenvironment signature; further *in vitro* and *in vivo* functional assays in subsequent sections are required to verify any potential causal regulatory mechanism linking PVR to CAR-T therapeutic resistance. Spearman’s correlation analysis showed that PVR expression was significantly correlated with multiple immune-related genes, including *PRARGC1A* (r = 0.022, *p* < 0.05) and *TCF7* (r = 0.037, *p* < 0.01, Figure 4D). These two genes are associated with T cell stemness and mitochondrial metabolism. Further validation using the TIMER 3.0 database confirmed that PVR expression was associated with signatures of CD4^+^ memory resting T cells and Th17 cells, rather than CD8^+^T cells and other immune cell subsets in ESCC (*n* = 184, Figure 4E,F). These results suggest that PVR may modulate the immune microenvironment and contribute to poor clinical outcomes in ESCC.

### 2.5. PVR Knockdown Enhances Anti-Tumor Efficacy of IL21.CD276.CAR-T Cells

**Figure 5:**
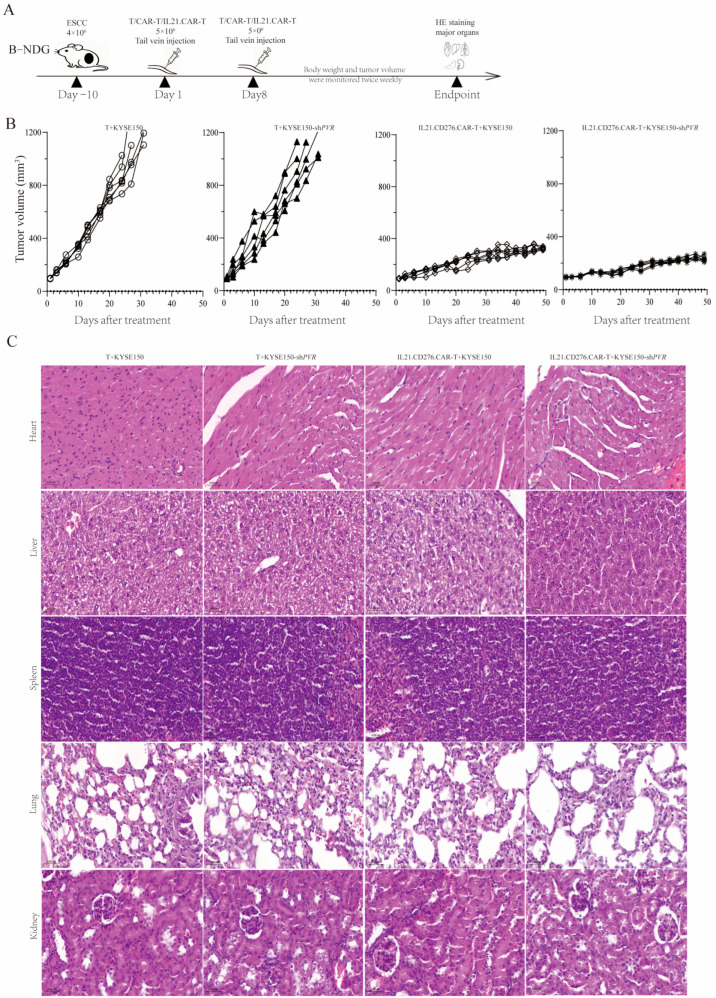
*In vivo* validation of PVR-mediated resistance to IL21.CD276.CAR-T therapy. (**A**) Schematic overview of the *in vivo* experimental. (**B**) Tumor growth curves in B-NDG mice bearing KYSE150 or KYSE150-sh*PVR* xenografts treated with T cells or IL21.CD276.CAR-T cells (*n* = 6, one-way ANOVA). Significantly reduced final tumor volume was detected in IL21.CD276.CAR-T-treated KYSE150-sh*PVR* group vs IL21.CD276.CAR-T-treated KYSE150 group, *p* < 0.05. (**C**) H&E staining of major organs from mice in each treatment group.

To validate the role of PVR in resistance to IL21.CD276.CAR-T therapy *in vivo*, we established B-NDG xenograft models using KYSE150 and KYSE150-sh*PVR* cells (Figure 5A). Tumor-bearing mice were treated with T cells or IL21.CD276.CAR-T cells. As shown in Figure 5B, following IL21.CD276.CAR-T treatment, KYSE150-shPVR-derived tumors exhibited a tendency toward delayed tumor progression compared with parental KYSE150 tumors. While tumor volumes partially overlapped during the early observtion stage, statistically significantly smaller terminal tumor sizes were observed in the KYSE150-shPVR group based on repeated-measures one-way ANOVA analysis. These data suggest that *PVR* knockdown may contribute to delayed tumor progression and reduced terminal tumor volume without complete tumor elimiation. To evaluate potential toxicity, major organs (heart, liver, spleen, lung and kidney) were harvested at the endpoint for H&E staining. No obvious histological abnormalities or signs of toxicity were observed in any of the treatment groups (Figure 5C). No significant changes in body weight were noted in mice during the treatment period (Figure S1C). These *in vivo* results confirm that *PVR* knockdown enhances the therapeutic efficacy of IL21.CD276.CAR-T cells without increasing systemic toxicity.

## 3. Discussion

Approved CD19.CAR-T has achieved remarkable durable complete remission rates for relapsed/refractory pediatric B-cell acute lymphoblastic leukemia, while therapeutic bottlenecks persist in pediatric T-cell acute lymphoblastic leukemia and acute myeloid leukemia owing to antigen loss and tumor heterogeneity. Despite these promising outcomes in hematological malignancies, CAR-T therapy has achieved encouraging progress in the treatment of solid tumors, but its clinical efficacy is severely limited by the emergence of therapeutic resistance [24,25]. Beyond insufficient anti-tumor efficacy, infectious complications represent another major clinical hurdle restricting broad CAR-T application across pediatric and young adult cohorts. Post-infusion immunosuppression from prior chemotherapy and CAR-mediated B-cell depletion predisposes patients to early bacterial/fungal sepsis and late recurrent viral/bacterial infections; incidence and dominant pathogen types differ between young children and young adult patients. This clinical complication background helps explain the clinical translational value of improving CAR-T anti-tumor efficacy while reducing off-target immune suppression in our study. CD276 is a promising target for ESCC CAR-T therapy owing to its high and specific expression in tumor tissues and relative lower expression in normal tissues [26]. We constructed an IL-21-armored CD276-targeted CAR-T, IL21.CD276.CAR-T, to enhance anti-tumor function. Although IL21.CD276.CAR-T exhibited stronger cytotoxicity against ESCC cells *in vitro* than classical CD276.CAR-T, it failed to induce complete tumor regression in B-NDG xenograft models, indicating the existence of prominent resistance mechanisms.

Resistance to CAR-T therapy is frequently attributed to antigen loss or downregulation [27,28]. However, our results demonstrated that CD276 expression remained stably high in recurrent tumor cells after IL21.CD276.CAR-T treatment, ruling out target loss as the main cause of resistance. Instead, we identified that the inhibitory checkpoint ligand PVR was significantly upregulated on ESCC cells after co-cultured with IL21.CD276.CAR-T. Moreover, baseline PVR expression varied among ESCC cell lines, and high endogenous PVR predicted poor sensitivity to CAR-T. These findings indicate that PVR upregulation acts as an adaptive immune escape mechanism in response to IL21.CAR-T-mediated immune pressure.

Notably, we confirmed that IL-21R was undetectable on all the three ESCC cell lines, excluding the possibility that IL-21 directly acts on cancer cells to induce PVR expression. This result clarifies that PVR upregulation is a passive adaptive response of tumor cells under CAR-T treatment, rather than a direct effect of IL-21 signaling. PVR is the main ligand of the inhibitory receptor TIGIT which is highly expressed on activated and exhausted T cells [29,30]. The binding of TIGIT on CAR-T cells to PVR on cancer cells can suppress cytotoxicity, promote exhaustion, and impair persistence, ultimately leading to therapy failure [31,32,33]. Our findings reveal a PVR-mediated resistance axis that restricts the efficacy of IL-21-armored CAR-T in ESCC. Due to the slightly lower upregulation of PVR expression in the tolerant environment, in order to rule out non-specific changes, we examined the consistency of EpCAM abundance before and after co-incubation of KYSE70 with T, CAR-T, IL21.CAR-T, and confirmed that the increase in PVR signal is a specific adaptive response of tumor cells to the stimulation of IL21.CD276.CAR-T, rather than a general increase in total cellular proteins.

Bioinformatics analysis from the TCGA cohort further supported the clinical significance of PVR. High PVR expression was associated with poor overall survival, reduced T-cell infiltration, and an immunosuppressive tumor microenvironment. In addition, PVR expression was negatively correlated with T-cell stemness and metabolic fitness–related genes such as *TCF7* and *PPARGC1A*, suggesting that PVR shapes a microenvironment that suppresses T-cell function and promotes exhaustion. These data highlight PVR as a prognostic biomarker and a promising therapeutic target for ESCC.

Functional validation experiments confirmed that shRNA-mediated *PVR* knockdown markedly restored the sensitivity of ESCC cells to IL21.CD276.CAR-T *in vitro*. More importantly, *in vivo* xenograft models showed that *PVR* knockdown significantly enhanced the anti-tumor function of IL21.CD276.CAR-T, with tumor control and no obvious systemic toxicity. These results provide preclinical proof-of-concept that simultaneously targeting CD276 and PVR can overcome resistance and improve the efficacy of IL-21 armored CAR-T therapy.

This study still has some limitations. First, the experiments were performed in immunodeficient mice, which cannot fully recapitulate the human immune microenvironment. Second, the upstream signaling pathway by which CAR-T pressure induces PVR upregulation remains to be explored. Third, the safety and efficacy of combinatorial strategies require further validation in clinical trials. Despite these limitations, our study indicates a role for PVR in mediating resistance to IL21.CD276.CAR-T therapy in ESCC.

## 4. Materials and Methods

### 4.1. Cell Lines and Culture

Human ESCC cell lines KYSE70, KYSE150, KYSE450 and Jurkat were cultured in RPMI 1640 (Gibico, Grand Island, NY, USA) medium supplemented with 10% fetal bovine serum (FBS, PAN SERATECH, Aidenbach, Germany). HEK-293T cells were cultured in DMEM (Gibico) medium and 10% FBS at 37 °C with 5% CO_2_. Human peripheral blood mononuclear cells (PBMC) were donated from healthy volunteers. Human T cells, CAR-T cells and IL21.CAR-T cells were cultured in X-vivo (Lonza, Walkersville, MD, USA) and 5% FBS and human IL-2 (PeproTech, Rocky Hill, NJ, USA, Cat 200-02-50 μg, 50–100 IU/mL). The study was approved by the ethics committee of Air Force Medical Center, PLA (License 2025-75-PJ01) and all donors had signed the informed consent.

### 4.2. Generation and Production of CAR-T Cells

The CD276.CAR was generated as reference [9]. The IL-21-armored CD276.CAR constructs were designed with the following structure: 5′-LTR-EF-1α-signal peptide IL-21-T2A-scFv-CD8-4-1BB-CD3ζ-3′-LTR. Lentiviral vectors were packaged in HEK-293T cells and lentiviruses were harvested in supernatants and stored at −80 °C. Jurkat cells were used for titer calculation. T cells were positively selected from PBMC using dynabeads human T-activator CD3/CD28 (Gibco, Grand Island, NY, USA, Cat 11141D). The dynabeads activated T cells were transduced with lentivirus with the MOI 1.5–2 on days 2 and 3 with SynperonicR F108 (Sigma-Alorich, St. Louis, MO, USA, Cat 07579) at 1500 g for 1.5 h at 30 °C (up 3 down 1). After repeated pre-exploration, MOI of 1.5–2 was selected to keep CAR positive proportion between 60% and 85%, with less than 10% variability among different donors and batches. The expression of CAR was detected by Protein L (Sino Biological, Beijing, China, Cat 11044H07EP, PE) by FCM before downstream experiments.

### 4.3. ELISA for IL-21 Secretion

T cells, CD276.CAR-T cells and IL21.CD276.CAR-T cells were cultured for 48 h, and the supernatant was collected. IL-21 concentration was measured using an ELISA kit (Invitrogen, Carlsbad, CA, USA, Cat 88-8218) according to the manufacturer’s instructions. ELISA measurements were performed with 3 independent biological replicates.

### 4.4. In Vitro Cytotoxicity Assay

The cytotoxic activity of T cells and CAR-T cells against ESCC cell lines was detected using a LDH release assay (DOJINDO, Kumamoto, Japan, Cat CK12). Tumor cells were digested by 0.025% EDTA, co-cultured with T or CAR-T cells at different E:T ratios (7.5:1, 5:1, 2.5:1, 1:1). The anti-tumor efficacy after 10 h of co-incubation was detected according to the manufacturer’s instructions. All cytotoxicity assays were performed with 3 independent biological replicates, and each biological replicate included 3 technical replicates per condition.

### 4.5. Flow Cytometry Analysis

The expression of CD276, PVR and IL-21R on ESCC cells, and CAR expression on CAR-T cells were analyzed by FCM using specific antibodies. Tumor cells were digested by 0.025% EDTA. After washed by PBS twice, cancer cells were incubated with CD276 antibody (PE, Biolegend, San Diego, CA, USA, Cat 331605) or IL-21R antibody (PE, Invitrogen, Carlsbad, CA, USA, Cat 12-3601-42) for 30 min at 4 °C in the dark. For construction of the *in vitro* CAR-T-resistant cell model, an E:T ratio of 1:1 was used to simulate the resistant environment of IL21.CAR-T cells for 48 h. After washing away the IL21.CAR-T cells, the expression of PVR (PE, Biolegend, San Diego, CA, USA, Cat 337609) on the membrane of KYSE70 cells was detected. Similarly, tumor cells were digested by EDTA, incubated with CD276 antibody in the dark for 30 min, and then analyzed by FCM. The CAR expression of CAR-T cells or IL21.CAR-T cells were detected by Protein L for 30 min at 4 °C. Data were analyzed by Flow Jo V10 software. Single intact cells were first gated by FSC-A vs. SSC-A, and cell doublets were excluded via FSC-H versus FSC-A gating. Tumor cell populations were subsequently gated according to cellular scatter features, with unified gating criteria applied for all samples. All flow cytometry experiments were repeated with 3 independent biological replicates, with at least 10,000 events acquired per sample.

### 4.6. PVR Knockdown Cell Lines

For the establishment of *PVR*-knockdown cell lines, shRNA targeting *PVR* and negative control shRNA were packaged into lentiviral particles, respectively. KYSE-150 and KYSE-450 cells were then infected with the lentivirus. After lentiviral infection 24 h, the culture medium was replaced, and cells were cultured in RPMI 1640 and 10%FBS with 0.5–2 μg/mL puromycin (Sigma Aldrich, St. Louis, MO, USA, Cat. P9620) for 10–14 days to screen *PVR* knockdown cell pools. FCM was used to detect and verify the knockdown efficiency of *PVR*. Subsequently, the single-cell clone with the most significant *PVR* silencing effect was isolated for subculture, large-scale expansion and cryopreservation.

### 4.7. In Vivo Xenograft Models

B-NDG mice (Biocytogen, Beijing, China) were subcutaneously inoculated with 4 × 10^6^ KYSE70, KYSE150, or KYSE150-shPVR cells. When tumors volume reached to nearly 100 mm^3^, mice were randomized to receive T cells, CD276.CAR-T, or IL21.CD276.CAR-T cells via tail vein injection on Day 1 and 8 (*n* = 6). Tumor volume and body weight were monitored twice a week by an investigator blinded to group allocation. At the endpoint (tumor volume reach to 1000 mm^3^), major organs were harvested for hematoxylin and eosin staining to evaluate toxicity. At the endpoint, tumors were harvested from B-NDG mice inoculated with KYSE70 cells and treated with IL21.CD276 CAR-T cells. The tumors were washed three times with PBS, minced into small pieces with a scalpel, and digested in a mixture including RPMI 1640, collagenase I (Solarbio, Beijing, China, Cas 9001-12-1), collagenase IV (Solarbio, Beijing, China, Cas 9001-12-1) and hyaluronidase (Solarbio, Cas 37326-33-3) with shaking at 37 °C for 1 h. Digestion was terminated with RPMI 1640 with 10% FBS. After centrifugation at 800 rpm for 5 min at room temperature, the pellets were cultured in DMEM/F12 medium (M&C Gene, Beijing, China, Cat CM10090) containing 10% FBS and antibiotics. The expression of CD276 on primary cancer cells (P0) was determined by FCM. These cells were then co-cultured with IL21.CD276.CAR-T cells *in vitro* at an E:T ratio of 2:1. The resistant cells (P1) were collected and detected by FCM again to evaluate CD276 antigen expression.

### 4.8. Hematoxylin and Eosin (H&E) Staining

Major organs including heart, liver, spleen, lung, and kidney were harvested from B-NDG mice treated with T, CD276.CAR-T or IL21.CD276.CAR-T cells, washed with PBS, and fixed in 4% paraformaldehyde for at least 24 h. Fixed tissues were dehydrated through a graded ethanol series, cleared in xylene, embedded in paraffin, and sectioned at about 5 μm. After deparaffinization and rehydration, sections were stained with hematoxylin for 5 min, differentiated in 1% hydrochloric acid alcohol, and blued in ammonia water. Sections were counterstained with eosin for 1 min, dehydrated again, cleared in xylene, and mounted with neutral balsam. Histological changes were observed and photographed under an optical microscope to evaluate systemic toxicity induced by the treatment.

### 4.9. Bioinformatics Analysis

Expression profiling and corresponding clinical survival data were obtained from The Cancer Genome Atlas (TCGA) database. We then extracted data in TPM format and performed normalization using the log2(TPM + 1) transformation.

Kaplan-Meier (KM) analysis was performed using the R package “survival package” (version 4.2.1) to test the proportional hazards assumption and to fit survival regression models (*n* = 184). Univariate Cox proportional hazards regression was performed to calculate the hazard ratio (HR) with a 95% confidence interval (CI) and the corresponding *p* value. ROC curves, with the area under the curve (AUC) and 95% CI were analyzed using the R package “pROC” (version 1.18.0) and the results were visualized with ggplot2 (version 3.4.4). To conduct a reliable immune score assessment, we used R package “immunedeconv” (version 4.1.3). We used the R package “ggClusterNet package” (version 4.2.1) for analysis and visualization of the results. Correlation analysis of PVR with immune-related genes and the immune cell signatures analyzed using the TIMER3.0 database (https://compbio.cn/timer3/, 5 February 2026). All data analyses and graphs were performed using R package “survival package” (version 4.2.1). *p* < 0.05 was considered statistically significant.

### 4.10. Western Blot

For Western blot detection of PVR (HUABIO, Hangzhou, Zhejiang, China, Lot H641512034), EpCAM (HUABIO, Hangzhou, Zhejiang, China, Lot H640161030) and pZAP70 (Proteintech, Rosemont, IL, USA, Cat 81767-2-RR), we established parallel co-culture systems to mimic the resistant environment as described in Section 4.5. Briefly, untreated KYSE70 cells, KYSE70 co-cultured with unmodified T cells, CD276.CAR-T cells or IL21.CD276.CAR-T cells were collected for tumor total protein extraction to detect PVR and EpCAM. Meanwhile, T cells, CD276.CAR-T cells, IL21.CD276.CAR-T cells cultured alone, as well as the three effector subsets after co-incubation with KYSE70 were harvested to extract cellular protein for pZAP70 measurement.

All cell pellets were lysed with RIPA lysis buffer (containing 1 mM PMSF, phosphatase inhibitor cocktail and protease inhibitor cocktail) on ice for 30 min. After centrifugation at 12,000× *g* for 15 min at 4 °C, the supernatant was collected, and total protein concentration was quantified using a BCA protein assay kit. Equal amounts of total protein from each group were separated via 10% SDS-PAGE gel electrophoresis and transferred onto PVDF membranes. Membranes were blocked with 5% non-fat milk diluted in TBST buffer at room temperature for 1 h, followed by incubation with primary antibodies against PVR, EpCAM, pZAP70 and GAPDH overnight at 4 °C. After three washes with TBST, membranes were incubated with corresponding HRP-conjugated secondary antibodies at room temperature for 1 h. Protein bands were visualized using enhanced chemiluminescence reagent on a chemiluminescence imaging system. Band gray values were quantified by ImageJ (version 1.54) software and normalized to the internal reference GAPDH for statistical comparison.

### 4.11. Statistical Analysis

All statistical analyses were performed using GraphPad Prism 8.0. Data are presented as mean ± standard deviation (SD). Survival curves were analyzed using the Kaplan-Meier method and log-rank test. Comparisons between groups were performed using Student’s *t*-test or one-way ANOVA. A *p*-value < 0.05 was considered statistically significant.

## 5. Conclusions

Our preclinical data indicate that elevated PVR expression is associated with impaired anti-tumor activity of IL21.CD276.CAR-T against ESCC and acts as a candidate factor driving therapeutic resistance. However, all *in vivo* results were obtained in immunodeficient B-NDG xenograft mice, which fail to recapitulate the intact human tumor immune microenvironment. Targeted suppression of PVR combined with IL21-modified CD276 CAR-T represents a promising preclinical combinatorial hypothesis for future ESCC treatment rather than an immediately available validated clinical regimen. Further work including identification of upstream regulators of PVR upregulation and repeated functional verification in immunocompetent or humanized animal models is indispensable to advance the translational potential of this therapeutic combination.

## Data Availability

The data presented in this study are available upon request from the corresponding author.

## Supplementary Materials

The following supporting information can be downloaded at: https://www.mdpi.com/article/10.3390/ijms27156725/s1.

## Abbreviations

The following abbreviations are used in this manuscript:

AUC	Area under the curve
CAR	Chimeric antigen receptor
CI	Confidence interval
ESCC	Esophageal squamous cell carcinoma
EGFR	Epidermal growth factor receptor
EpCAM	Epithelial cell adhesion molecule
E:T	Effector-to-target
FCM	Flow cytometry
HR	Hazard ratio
IL-21	Interleukin-21
ITIM	Immunoreceptor tyrosine-based inhibitory motif
GPC3	Glypican-3
MHC	Major histocompatibility complex
MOI	Multiplicity of infection
NKG2D	Natural killer group 2D
PBMC	Peripheral blood mononuclear cells
PVR	Poliovirus receptor
pSHP2	Phospho-Src homology 2 domain-containing phosphatase 2
pZAP70	Phospho-zeta-chain-associated protein kinase 70
ROC	Receiver operating characteristic
SD	Standard deviation
TCGA	The Cancer Genome Atlas
TIP	Tumor immunophenotype
Tfh	Follicular helper T cell
TIGIT	T Cell Immunoglobulin and ITIM Domain
