# Supplementary material for: PVR Mediates Resistance to IL21.CD276.CAR-T Therapy in Esophageal Squamous Cell Carcinoma
Source: Int J Mol Sci. 2026 Jul 28;27(15):6725. doi: 10.3390/ijms27156725 (PMC13466472; doi:10.3390/ijms27156725)
Supplement: Supplementary file 1 [file ijms-27-06725-s001.zip › ijms-4317413-supplementary.pdf]

A

PVR-sh1-F:CCGGCCCAGCTATTCGGAGTCCAAActcgagTTTGGACTCCGAATAGCTGGGTTTTTG

PVR-sh1-R:aattCAAAAACCCAGCTATTCGGAGTCCAAActcgagTTTGGACTCCGAATAGCTGGG

PVR-sh2-F:CCGGGAGGTATCCATCTCTGGCTATctcgagATAGCCAGAGATGGATACCTCtttttg

PVR-sh2-R:aattcaaaaaGAGGTATCCATCTCTGGCTATctcgagATAGCCAGAGATGGATACCTC

PVR-sh3-F:ccggCAGAGCCCACAGGCTATAATTctcgagAATTATAGCCTGTGGGCTCTGtttttg

PVR-sh3-R:aattcaaaaaCAGAGCCCACAGGCTATAATTctcgagAATTATAGCCTGTGGGCTCTG

B

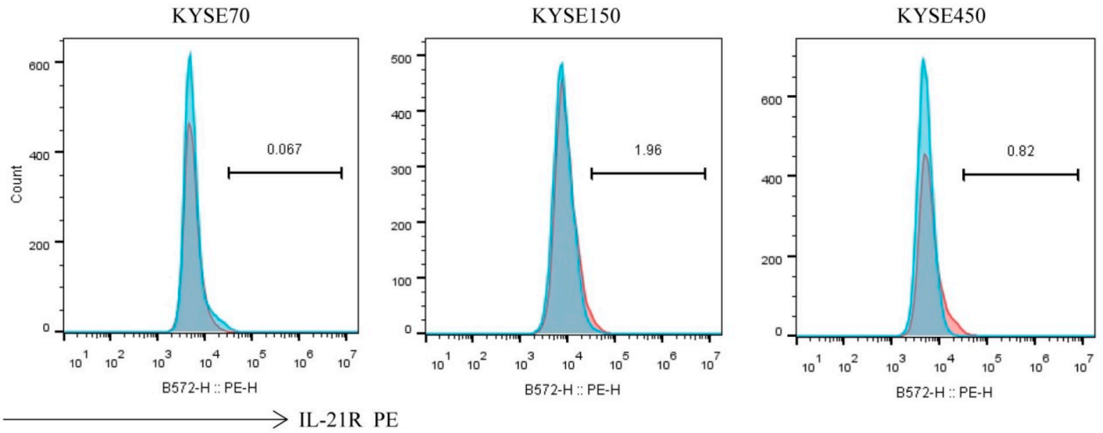

C

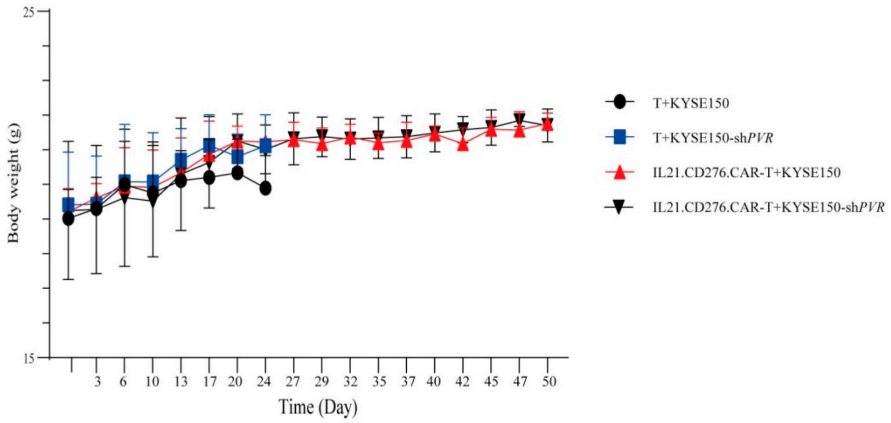

Figure S1 Sequences of shRNA constructs targeting human PVR, flow cytometric analysis, and body weight changes of mice during treatment. A Sequences of three independent shRNA constructs targeting human PVR. B Flow cytometry analysis of IL-21R expression on KYSE70, KYSE150, and KYSE450 cells. C Body weight changes of B-NDG mice in each treatment group (n=6).
